# Development of stereo endoscope system with its innovative master interface for continuous surgical operation

**DOI:** 10.1186/s12938-017-0376-1

**Published:** 2017-06-24

**Authors:** Myungjoon Kim, Chiwon Lee, Nhayoung Hong, Yoon Jae Kim, Sungwan Kim

**Affiliations:** 1 Interdisciplinary Program for Bioengineering, Graduate School, Seoul National University, Seoul, 03080 South Korea; 2 Korea Electrotechnology Research Institute, Ansan, 15588 South Korea; 3 Institute of Medical and Biological Engineering, Seoul National University, Seoul, 08826 South Korea; 4 Department of Biomedical Engineering, Seoul National University College of Medicine, Seoul, 03080 South Korea

**Keywords:** Three-dimensional (3D) endoscope system, Minimally invasive surgery (MIS), Improved novel master interface (iNMI), da Vinci research kit (dVRK), Hands-on-throttle-and-stick (HOTAS)

## Abstract

**Background:**

Although robotic laparoscopic surgery has various benefits when compared with conventional open surgery and minimally invasive surgery, it also has issues to overcome and one of the issues is the discontinuous surgical flow that occurs whenever control is swapped between the endoscope system and the operating robot arm system. This can lead to problems such as collision between surgical instruments, injury to patients, and increased operation time. To achieve continuous surgical operation, a wireless controllable stereo endoscope system is proposed which enables the simultaneous control of the operating robot arm system and the endoscope system.

**Methods:**

The proposed system consists of two improved novel master interfaces (iNMIs), a four-degrees of freedom (4-DOFs) endoscope control system (ECS), and a simple three-dimensional (3D) endoscope. In order to simultaneously control the proposed system and patient side manipulators of da Vinci research kit (dVRK), the iNMIs are installed to the master tool manipulators of dVRK system. The 4-DOFs ECS consists of four servo motors and employs a two-parallel link structure to provide translational and fulcrum point motion to the simple 3D endoscope. The images acquired by the endoscope undergo stereo calibration and rectification to provide a clear 3D vision to the surgeon as available in clinically used da Vinci surgical robot systems. Tests designed to verify the accuracy, data transfer time, and power consumption of the iNMIs were performed. The workspace was calculated to estimate clinical applicability and a modified peg transfer task was conducted with three novice volunteers.

**Results:**

The iNMIs operated for 317 min and moved in accordance with the surgeon’s desire with a mean latency of 5 ms. The workspace was calculated to be 20378.3 cm^3^, which exceeds the reference workspace of 549.5 cm^3^. The novice volunteers were able to successfully execute the modified peg transfer task designed to evaluate the proposed system’s overall performance.

**Conclusions:**

The experimental results verify that the proposed 3D endoscope system enables continuous surgical flow. The workspace is suitable for the performance of numerous types of surgeries. Therefore, the proposed system is expected to provide much higher safety and efficacy for current surgical robot systems.

## Background

A minimally invasive surgery (MIS) has been introduced with advances in medical technologies and recently diverse technologies have improved MIS techniques such that the MIS is currently one of the most encouraged surgical operation approaches nowadays. Although conventional MIS offers several advantages [1, 2], it still suffers from below weaknesses: (i) longer operation time than conventional open surgery and (ii) surgical instruments with low degrees of freedom (DOF), which results in longer learning curve for complex surgical operation; thus, extensive training is needed to perform surgical operations [3–5]. Robotic laparoscopic surgery was subsequently introduced to overcome the limits of both kinds of surgeries and provide various benefits [6–10].

The da Vinci surgical robot (Intuitive Surgical Inc., Sunnyvale, CA, USA), which is the most cutting-edge and predominant surgical robot, has been used in more than three million MIS operations worldwide since 2000 [11]. Nevertheless, several issues still remain to be resolved. Among these issues is the discontinuous surgical flow that occurs whenever control is switched between the endoscope system and the patient side manipulator (PSM), which also exists in various surgical robot systems including the da Vinci surgical robot system. In this scenario, the surgeon has to abandon the control of the PSM by using a clutch button or pedal in order to control the endoscope system to change his/her view. The same control technique is required to regain control of the PSM [12]. This maneuver can lead to problems such as collision between surgical instruments, injury to patients (from surgical instruments being out of sight), increased operation time, and having to endure an unsatisfactory view to avoid swapping control [13]. Various approaches that attempt to solve this discontinuity issue proposed new master interfaces to control the endoscope system in parallel with the PSM. For example, a novel human–machine interface that tracks the surgeon’s facial motion via his/her iris and a tracker placed on his/her forehead has been proposed to control the position of the laparoscope [14]. A command interface for a combination of mouth gesture and voice command has also been proposed to control 3-DOF robotic endoscope systems [12]. An eye tracking endoscope control method has also been presented [15, 16] and a voice controlled robotic endoscope holder was developed [16]. Further, an interface that utilizes a pressure sensor sheet to track foot movement has been used to control surgical robot tools [17].

However, although the proposed interfaces allow simultaneous control of the endoscope system and the PSM, they also have limitations: (i) they cannot be adapted to current robot-assisted surgical systems as the surgeon’s head and foot are already occupied [14, 17], (ii) they prevent surgeons from giving verbal orders to assistants, which is essential during surgical operations, as they use mouth and voice for control [12, 16], and (iii) they are prone to erroneous and unintended endoscopic movements that can in turn result in greater harm to patients [16, 18].

In this study, a surgical robot system is proposed to overcome these limitations with the objective of enabling continuous surgical operation by allowing simultaneous control of the PSM and the endoscope system. The system consists of a da Vinci research kit (dVRK), a 4-DOFs endoscope control system (ECS), a simple three-dimensional (3D) endoscope, and two improved novel master interfaces (iNMIs). The dVRK is used as operation robot system, which is a research kit donated by Intuitive Surgical, Inc., and includes master tool manipulator (MTM), PSM, a foot pedal, and a stereo viewer from the first generation of the da Vinci surgical robot system. Since the endoscope system of da Vinci surgical robot is not included in the dVRK system, the 4-DOFs endoscope system and the simple 3D endoscope are developed to provide stereo view to the surgeon. The 4-DOFs ECS, developed to control the position of the endoscope, consists of four servo motors, which facilitate pitching, yawing, rolling, and translational motions, and a two-parallel link structure that enables stable fulcrum point motion essential for laparoscopic surgery. The simple 3D endoscope consists of two complementary metal–oxide–semiconductor (CMOS) camera modules that provide real-time stereo view to the surgeon via the stereo viewer of dVRK. The original images obtained by the sensors undergo a stereo calibration and stereo rectification process that results in stereo vision. The outer diameter of the developed endoscope is 10 mm and each CMOS module is capable of generating images with a resolution of 640 × 480 pixels. The iNMI is proposed and developed to enable simultaneous control of the PSM and the endoscope system in order to eliminate the possibility of surgical instruments being out of sight and therefore prevents collision between surgical instruments and injury to patients. Furthermore, this will also shorten the surgical operation time, which could result in reduced surgeon fatigue according to previous research [19]. The iNMI is a wireless communication interface that enables intuitive control of the endoscope system, in this study the 4-DOFs ECS, and is an improved version of the novel master interface developed in our previous study [20]. The iNMI is based on the hands-on-throttle-and-stick (HOTAS) controller used broadly in flight control, and which was previously presented [2, 21]. In this study, a capacitive touch sensor array was developed and a wireless microprocessor used to intuitively reflect the surgeon’s decision. In addition, the size of the iNMIs is small enough to be easily installed to the MTMs of the dVRK system to maximize convenience to the surgeon when using the iNMIs to manipulate the ECS simultaneously with the MTM. In this sense, a surgeon is able to operate the endoscope system by manipulating the iNMI with index finger, which is currently only used for manipulating the finger clutch of da Vinci surgical robot system, and simultaneously control the MTM for operating the PSMs. Therefore, the continuous surgical flow is enabled. Multiple experiments were performed to evaluate the iNMI performance in terms of accuracy, latency, and power consumption. Further, modified peg transfer tasks which require adjustment of field of view throughout the tasks were also carried out to evaluate its clinical applicability and ease of use. The effectiveness and utility of the proposed stereo endoscope system with its master interface is then evaluated.

## Methods

To overcome the above-mentioned limitations, a novel surgical robot system consisting of the following four parts was developed: (i) a simple 3D endoscope that provides 3D vision to the surgeon via a stereo viewer, (ii) a 4-DOFs ECS to control the position of the developed endoscope, (iii) a dVRK-based operating robot system, and (iv) two iNMIs for the surgeon to intuitively control both the 4-DOFs ECS and the PSM simultaneously.

**Fig. 1:**
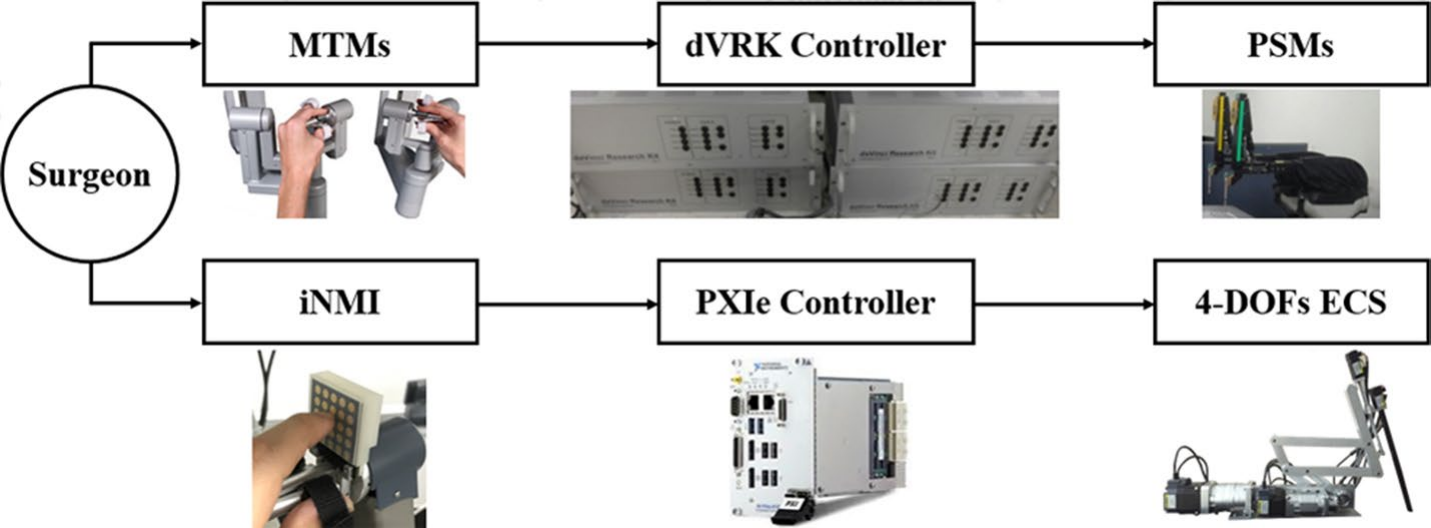
Control flow of the proposed surgical robot system driven by the surgeon’s intention. Software integration is based on the LabVIEW^Ⓡ^ software

The proposed system was integrated based on the PXIe controller and LabVIEW^Ⓡ^ (PXIe-8135 & 1062Q, LabVIEW^Ⓡ^ 2013, National Instruments, Austin, TX, USA), except for the dVRK system. Figure 1 illustrates the integrated system’s control flow. As illustrated in the figure, simultaneous operation of both PSMs and the developed 4-DOFs ECS is enabled via the two MTMs and the two iNMIs. Discontinuous surgical flow can then be overcame. The rolling motion, pitching motion, yawing motion, and translational motion of the 4-DOFs ECS are facilitated by four servo motors (Ezi-Servo Series, Fastech, Bucheon, South Korea).

### The simple 3D endoscope

To provide 3D vision to the surgeon and thereby ensure safe robotic surgery, a simple 3D endoscope, which is not included in the dVRK system, was developed explicitly for the laparoscopic surgical robot system. This simple 3D endoscope consists of two CMOS camera modules with six built-in light-emitting diodes (LEDs) and is capable of generating images with a resolution of 640 × 480 pixels, aligned in parallel to procure 3D vision. However, because simply aligning the two image sensors physically does not eliminate distortion or misalignment between the two camera modules, the two acquired images undergo a stereo calibration process, in which the geometric relationship between the two image sensors is calibrated to place them on the same plane, and a stereo rectification process that places the two calibrated images on a common image plane. These processes provide precise 3D vision to the surgeon via the stereo viewer. More specifically, the stereo calibration process includes the homography-based and chessboard calibration methods. The former method produces multiple sets of extrinsic parameters and enables calculation of the image sensor’s unique intrinsic and distortion parameters [22]. The latter method further improves the result by using chessboard [22]. Subsequently, stereo rectification is performed to horizontally align the two images and crop the effective image area, after which the reconstructed images are sent to the stereo viewer [23].

**Fig. 2:**
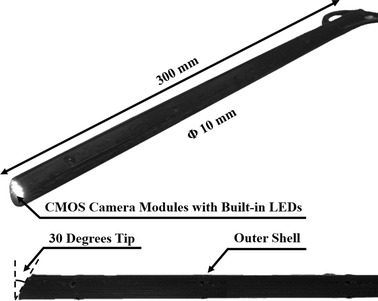
Simple three-dimensional (3D) endoscope manufactured using 3D printing technique. Two complementary metal–oxide–semiconductor (CMOS) camera modules are used for reconstructing stereo view. 6 Built-in light-emitting diodes (LEDs) of each module is used as light source. The tip of the endoscope is developed to have 30° to procure a wide range of view. The length and the diameter of the surgical instrument is designed as 300 and 10 mm, respectively

Through the above processes, the surgeon obtains 3D vision via the stereo viewer based on the calibrated and rectified images. In addition, the tip of the developed endoscope is bent at an angle of 30° to enable a wide vision range [24]. The outer case of the simple 3D endoscope is 10 mm in diameter and was manufactured based on rapid prototyping techniques (Form 1+ , Formlabs, Somerville, MA, USA) with resolution to the nearest millimeter, as shown in Fig. 2.

### The endoscope control system

**Fig. 3:**
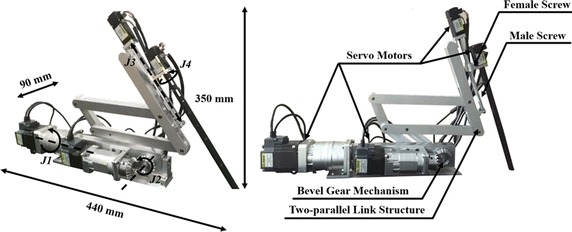
4-degrees of freedom (DOFs) endoscope control system (ECS). The fulcrum point motion is achieved by *J1* and *J2* with its two-parallel link structure. The translational motion and rolling motion are accomplished by *J3* and *J4*, respectively

The 4-DOFs ECS with four controllable joints, *J1* to *J4,* controls the position of the simple 3D endoscope, as shown in Fig. 3. It uses four servo motors to provide the 4-DOFs, comprising rolling motion, pitching motion, yawing motion, and translational motion, to the ECS. Furthermore, a two-parallel link structure was adopted to control the position of the developed 3D endoscope with optimized fulcrum point motion, which is necessary for laparoscopic robotic surgery. Thus, the fulcrum point motion of the system is guaranteed by its hardware structure, not by a control algorithm. This means that it is able to provide a reliable and fixed fulcrum point motion.

As shown in the figure, the yawing motion of the 3D endoscope is provided by *J1* and the pitching motion by *J2* based on the bevel gear mechanism that transmits rotational motion at a 90° angle. The translational motion is generated by *J3*. More specifically, it is accomplished by male and female screw mechanism which converts the rotation motion to the translational motion. Actuation of the motor of *J3* rotates the male screw and consequently the female screw moves translationally along two parallel line guides placed on either sides of the male and female screw arrangement. The rolling motion is provided by *J4*, which connects the motor’s shaft and the 3D endoscope via a customized coupler. Thus, the 4-DOFs ECS can perform the translational and fulcrum point motion during surgery by receiving the surgeon’s iNMI control.

### The da Vinci research kit

**Fig. 4:**
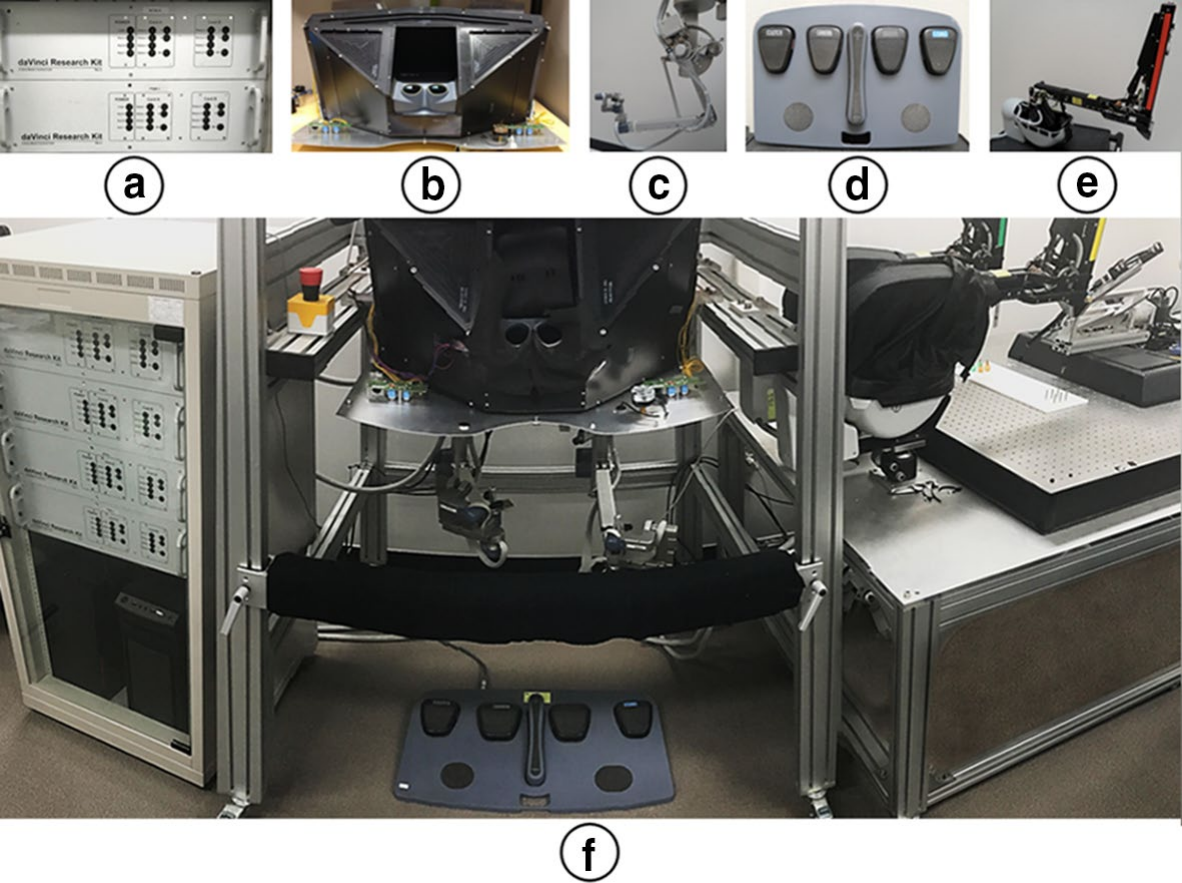
Overall system of the da Vinci research kit (dVRK). **a** Controllers. **b** Stereo viewer. **c** Master tool manipulators (MTMs). **d** Foot pedal. **e** Patient side manipulators (PSMs). **f** Installed dVRK. dVRK is used as operation surgical robot system in this research

The dVRK system is used as an operating surgical robot system, as shown in Fig. 4. It comprises two PSMs, two MTMs, one foot pedal, and one stereo viewer—which provides 3D stereo view constructed using stereo calibrated and rectified images obtained from the developed 3D endoscope. The two PSMs of the system can be operated using the two MTMs respectively during laparoscopic robotic surgery. The developed 4-DOFs ECS and the 3D simple endoscope are integrated with the dVRK system.

### The improved novel master interface

**Fig. 5:**
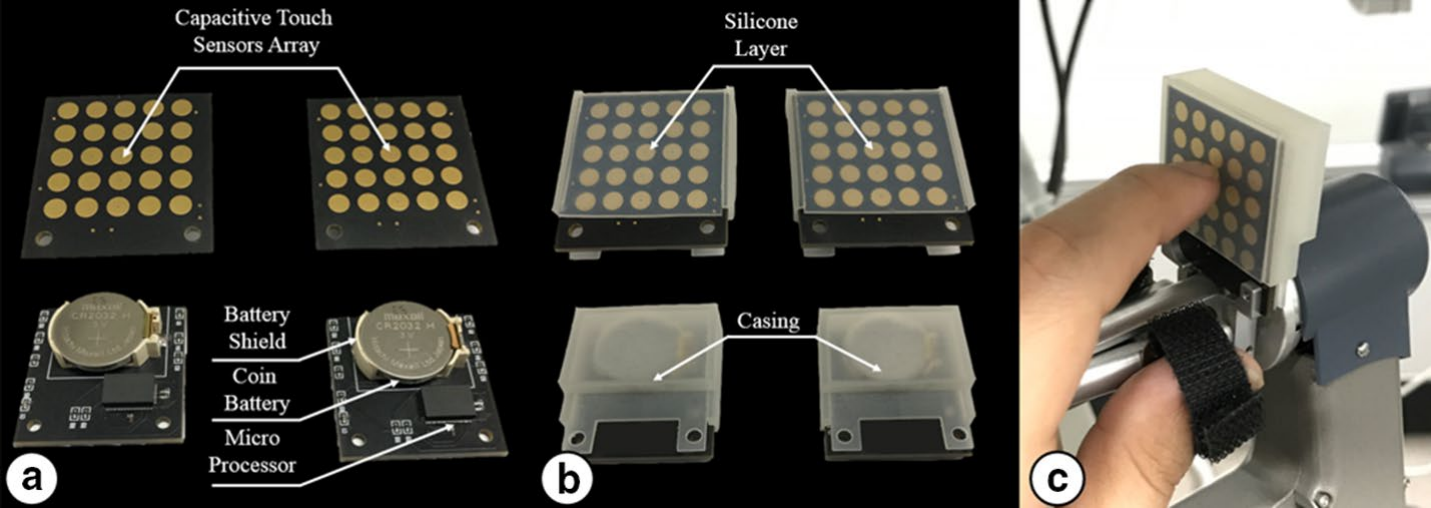
Developed improved novel master interface (iNMI). **a** Front and back sides of the iNMI. **b** Case and silicone *top layer* to protect the iNMI. **c** The iNMI attached on the MTM of the dVRK system using the special holder

The iNMI, which is a wireless communication interface, intuitively reflects the surgeon’s decision as regards control of the ECS. The iNMI was designed based on the HOTAS concept [2, 20], in which a multi-way switch is employed. To more intuitively reflect the surgeon’s intent, the iNMI utilizes a capacitive touch sensor array, based on a resistor–capacitor (RC) circuit with the capacitor as the touch sensor [25], instead of a multi-way switch. When the user touches the touch sensor, the RC time constant increases as the human body can be regarded as a relatively large capacitor [26]. Further, as the RC time constant can be calculated by setting a new state to the input of the RC circuit and then waiting for the output to be changed to the same state as the input, the touch status of the touch sensor can be determined. The gesture information generated from using the iNMI is obtained via the touch sensors array placed on the front of the iNMI, which comprises 25 capacitive touch sensors, as shown in Fig. 5a. The intent of the surgeon is determined from the touch status of the capacitive touch sensors array. In addition to the capacitive touch sensors array, the iNMI comprises one Arduino-based microprocessor with a Bluetooth low energy radio frequency module (RFD 77101, RFduino, Hermosa Beach, CA, USA), one Li–MnO_2_ type Lithium button cell battery (CR2032, Panasonic, Osaka, Japan), and several resistors and capacitors to complete the circuit.

**Fig. 6:**
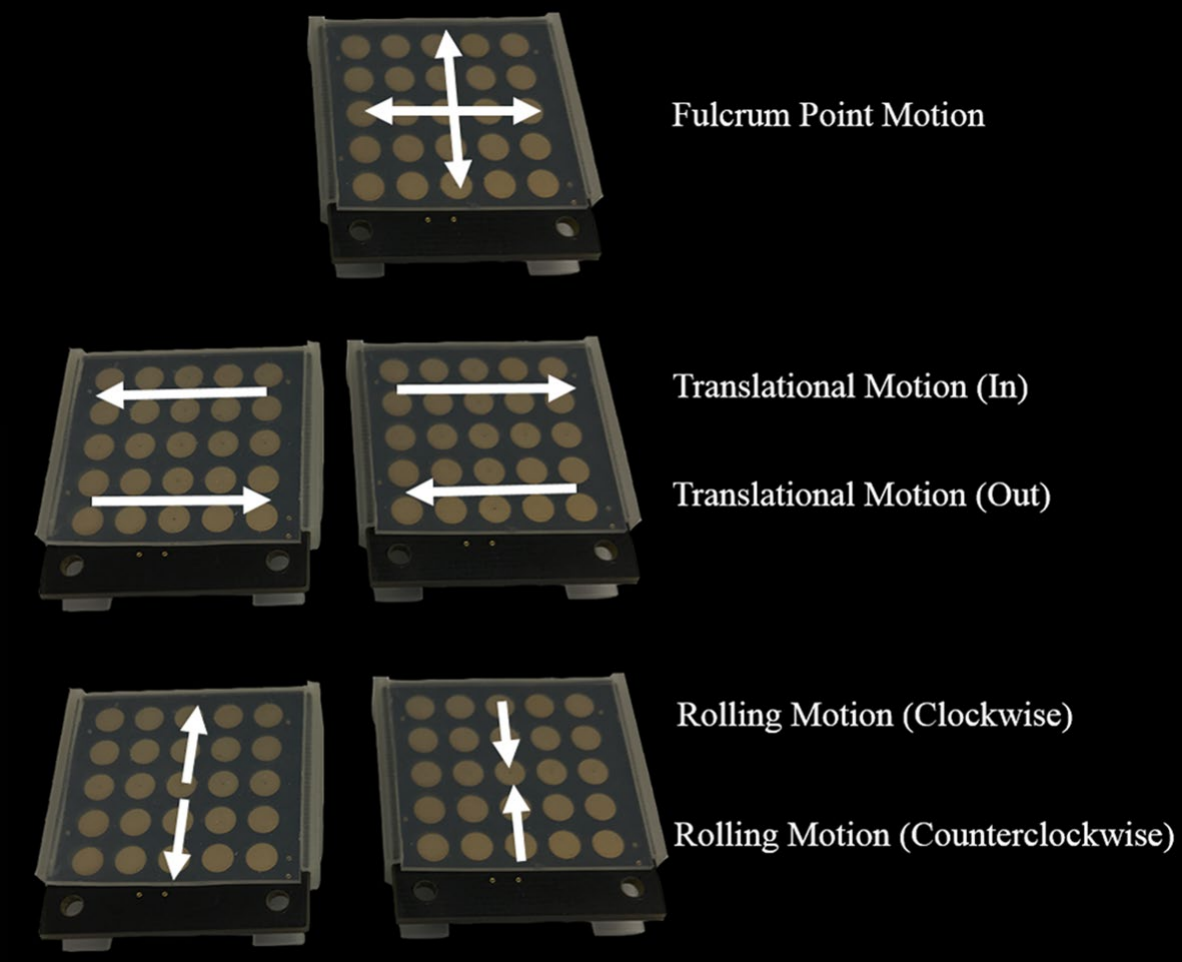
Mapping information between the iNMI and the 4-DOFs ECS. The fulcrum point motion can be achieved using one of the two iNMIs while the translational motion and rolling motion can only be performed by combination of two iNMIs’ gesture input

Each capacitive touch sensor is connected to the microprocessor to receive the RC time constant for perceiving the touch status of the capacitive touch sensor. To interpret the received touch information of the touch sensor array, a specific Arduino algorithm was developed to detect gesture information generated by the surgeon, such as upward swipe, downward swipe, forward swipe, and backward swipe. Once the gesture information is created, the Bluetooth module sends the information to the wireless data receiver, and the received signal is recognized as a command by the controller to manipulate the 4-DOFs ECS. To achieve above functions, a circuit was designed and a printed circuit board including the capacitive sensor array was consequently manufactured. Several parts were used for the board as shown in Fig. 5a. Further, it allows the surgeon to control the iNMI using the index finger that is not used to manipulate the MTM, but only to operate the finger clutch. Each iNMI has dimensions of 33 mm × 35 mm to ensure that they do not disrupt the motion of the MTMs. Furthermore, in order to protect the iNMI’s circuit and the capacitive touch sensor array, a customized outer case was designed and manufactured using a 3D printer and the touch sensor array is covered with a silicone layer, as shown in Fig. 5b. To manipulate the iNMIs simultaneously with the MTMs, the two iNMIs are tightly installed to the two MTMs of the dVRK system using a customized holder, as shown in Fig. 5c. In this way, the operation of the finger clutching the MTM is not interrupted, as occurs when using the da Vinci Si system [27]. Figure 6 shows the mapping information between the iNMI and the 4-DOFs ECS. As can be seen in the figure, the pitching motion and the yawing motion can be controlled by one of the two iNMIs, whereas the rolling motion and the translational motion can only be achieved by a combination of gestures generated by the iNMIs attached on the left and right MTMs. The mapped motions were deliberately designed to be similar to common touch gestures carried out in daily living activities using two fingers to ensure intuitive control by the surgeon. Thus, the pitching, yawing, rolling, and translation motions of the 4-DOFs ECS can be simultaneously controlled with PSMs by manipulating the two iNMIs and MTMs.

## Results

### The simple 3D endoscope

**Fig. 7:**
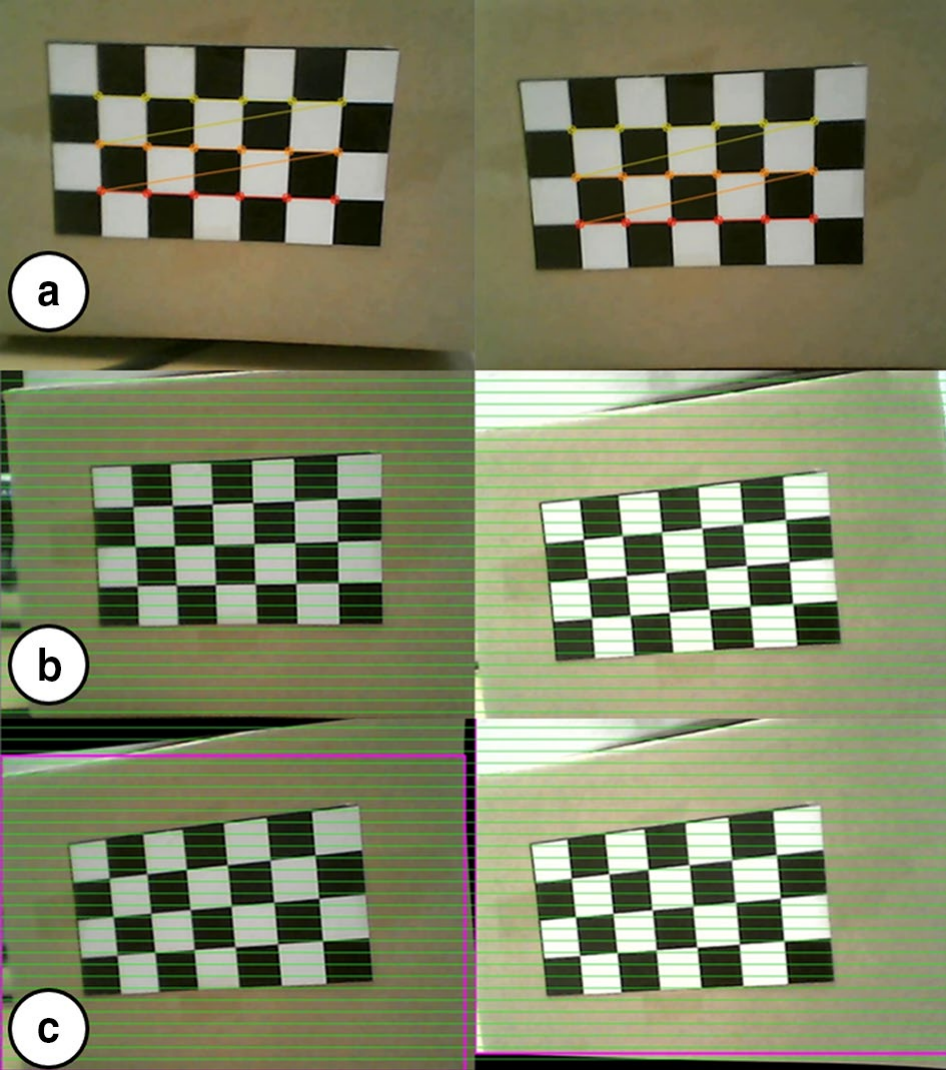
Stereo calibration and rectification processes. **a** Stereo calibration process. **b** Stereo rectification process. **c** Calibrated and rectified images with effective area enclosed in a *pink box*

Figure 7 shows the results of the stereo calibration and rectification process using the chessboard. As shown in Fig. 7a, the original images obtained by the two image sensors are distorted and misaligned, which means that the surgeon would not be able to comprehend them if they were simply projected onto the stereo viewer. Therefore, the stereo calibration process calculates intrinsic parameters such as distortion coefficients and then the two images are calibrated, as shown in Fig. 7b.

The stereo rectification results can be seen in Fig. 7c. The green lines in the figure are horizontal lines in the images for aligning the two images to the same height. The pink boxes represent the effective image area for stereo view. The effective image area of the obtained images are cropped and reconstructed for providing a clean stereo view to the surgeon.

**Fig. 8:**
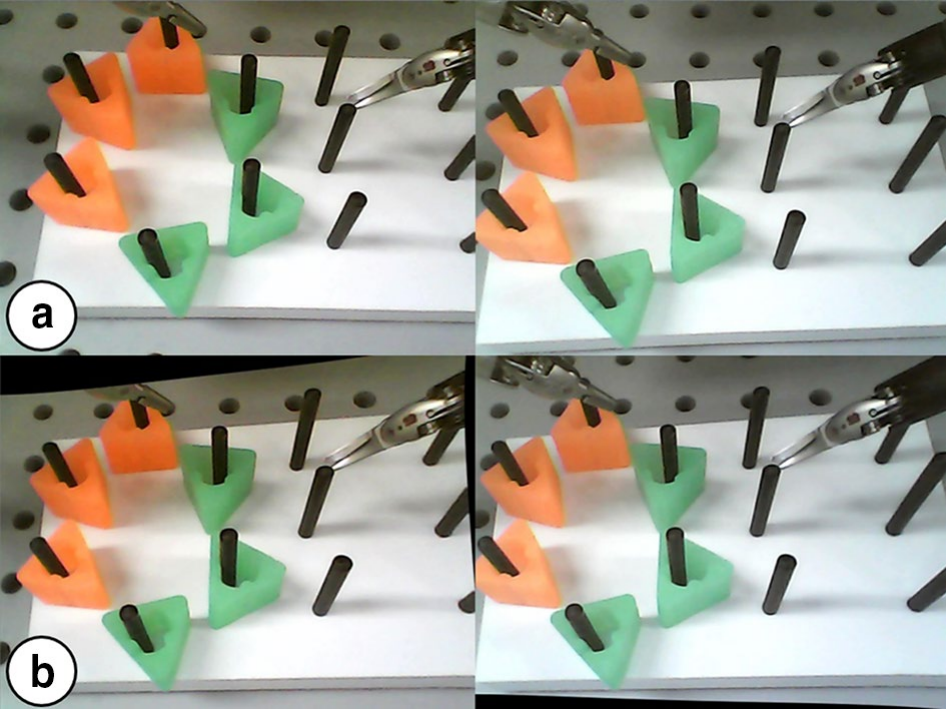
Comparison between original images and final images. **a** Original images obtained. **b** Final images after stereo calibration, rectification, and reconstruction

Figure 8 shows the original images and the final images provided to the surgeon after above processes.

### The endoscope control system

Table 1 summarizes the system specifications for the developed 4-DOFs ECS.

**Table 1:** System specifications of the 4-DOFs ECS

Specification item	Unit	Joints	
Installing posture			Floor mounted
Construction			Two-parallel link structure
Degrees of freedom			4
Drive method		J1–J4	Closed loop stepping system
Operation range	˚	J1	±45
		J2	±60
		J4	±360
	mm	J3	160
Maximum speed	rpm	J1, J2, J4	3000
	mm/s	J3	50
Resolution	/rev.	J1, J2	10,000
		J4	4000
	/mm	J3	4000
Entire system’s workspace	cm^3^		20378.3
Master interface			iNMI
Sterilization			Not available

*DOF* degrees of freedom, *ECS* endoscope control system, *iNMI* improved Novel master interface

### The improved novel master interface

#### Accuracy test

A specific LabVIEW^Ⓡ^ algorithm, which is able to receive the coordinate information of touched sensor in the array and the generated gesture information, was developed to estimate the precision of the iNMI. This was then achieved by acquiring the data generated by the iNMI which includes touch status of 25 touch sensors in total and the generated gesture motion for controlling the endoscope system, such as upward swipe, downward swipe, forward swipe, and backward swipe. The coordinate of touched sensor and gesture information were tested separately and repeated 50 times. Then, every signal was checked to verify whether the iNMI is able to correctly reflect user’s intent or misinterpret. No error detected during the repeated tests, which indicates that the iNMI is able to receive the surgeon’s decision with high accuracy.

#### Data transfer time

To evaluate the latency of the iNMI, the data generated by the iNMI were transferred by both wired communication and wireless communication. Wired communication was used as the reference data transfer time and acquired by physically connecting the iNMI using the universal serial bus port 2.0 type A. Therefore, the data transfer times of both types of communication were recorded using a specific LabVIEW^Ⓡ^ algorithm. Then, the difference between the arrival times of two data transferred by wired and wireless communication, which can be regarded as latency due to the iNMI’s wireless communication, was calculated. During the test, gesture motion was generated and sent by the iNMI. Furthermore, the gesture information has been encoded into a 1-byte number for minimizing the data transfer time. The latencies were measured for 50 times and the analysis of the results show that the latency of the iNMI was 5 ms in average, with a standard deviation (SD) of 1 ms.

#### Power consumption

Since the iNMI is a wireless master interface and therefore only operates based on wireless communication, it is crucial to secure enough power capacity to ensure its effectiveness and full utility during the surgery. To evaluate the power consumption, a LabVIEW^Ⓡ^ algorithm was developed to continuously intercept the data produced by the iNMI and record the time when there is no any data received, which indicates the iNMI was out of power. Although the power consumption of the iNMI can be calculated theoretically, it was tested using the above algorithm in order to practically evaluate the duration time of the iNMI. The tests were performed for 10 times. The results indicate that the iNMI is able to operate for 317 min (SD: 12 min), which is much longer than the average time for several types of robotic surgeries [1, 4, 28, 29]. However, the iNMI can also be used for surgeries which exceed its time duration since the battery can be simply changed to a new one which would cause minimum inconvenience. This implies that the iNMI, which is a wireless master interface and have its own easily replaceable power source, is able to be used for various type of surgeries. The iNMI is safe even when the iNMI battery has no power because it would not send any data that can control the ECS.

### Workspace

**Fig. 9:**
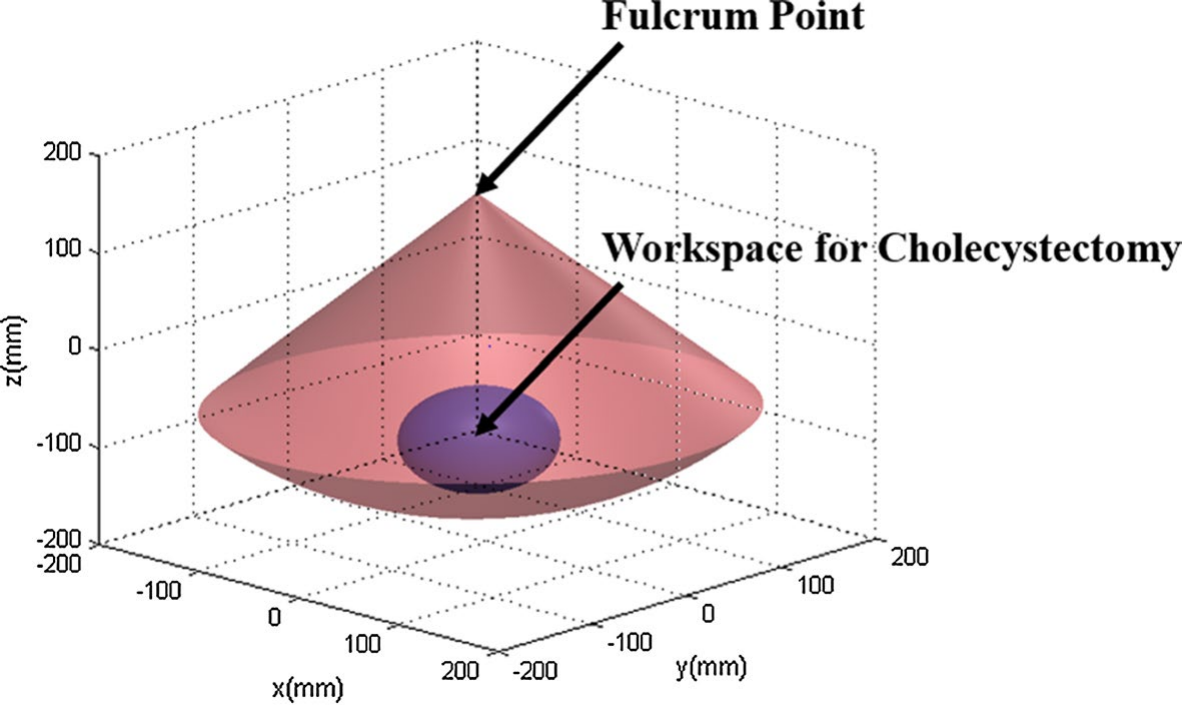
Workspace of the proposed 4-DOFs ECS

The workspace of the proposed ECS was calculated based on the hardware design of the system, and compared with the workspace required for cholecystectomy, as shown in Fig. 9. The calculated workspace is 20,378.3 cm^3^, which exceeds the reference workspace [30] of 549.5 cm^3^.

### Modified peg transfer task

**Fig. 10:**
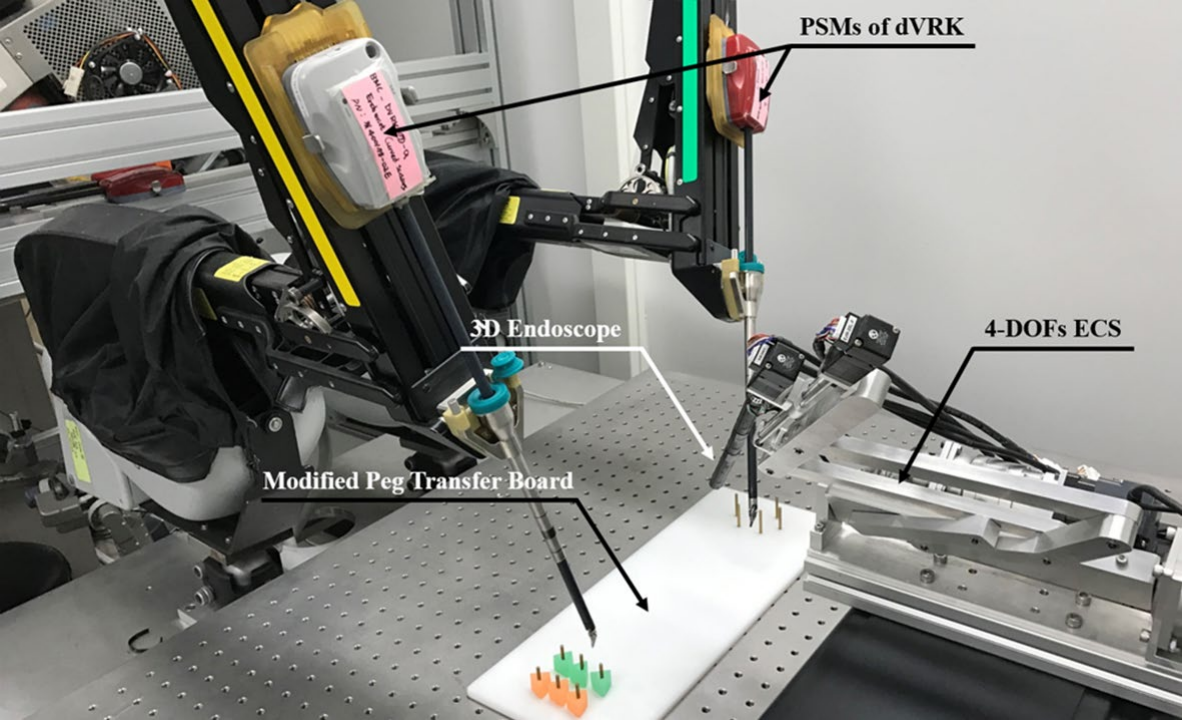
System setup for the modified peg transfer task. Modified peg transfer board was developed and used for the task to evaluate the overall performance of the proposed system

To validate the overall performance of the proposed laparoscopic surgical robot system, a peg transfer task was designed and performed using a newly developed peg transfer board redesigned based on the fundamentals of the laparoscopic surgery (FLS) peg transfer kit, as shown in Fig. 10. The peg transfer board was modified because the standard FLS peg transfer board does not require the endoscope system to be manipulated owing to its relatively small size, and therefore it is not able to evaluate the proposed surgical robot system consisting of the iNMI, simple 3D endoscope, and ECS, which requires manipulation of the endoscope system. Then, the FLS peg transfer task curriculum, which has already been defined in previous research for validation of surgical robot systems and measurement of the surgeon’s technical skills and eye-hand coordination during surgery [2, 20, 31, 32], was performed using the new peg transfer board. Three novice volunteers were recruited for the tasks and followed next steps: (i) the volunteers were asked to transfer six objects from the left side of the board to the right side of the board and (ii) the time taken to transfer the six objects, between the volunteer picking up the first object and releasing the last object, was measured. In addition, during the task, the volunteers were requested to always ensure that all surgical instruments were in view to mimic an actual surgical environment where safety has to be ensured. Furthermore, this is also one of the standards to evaluate the robotic surgical skills of the surgeon. The number of surgical instruments obscured from view was also counted during the experiments. The system setup is shown in Fig. 10. The stereo viewer was used for the task and the obtained calibrated and rectified images projected onto the stereo viewer to provide 3D vision to the users. Same with other studies, the time limit of the experiment was also set to 300 s in spite of the relatively long peg transfer board and use of the stereo viewer [2, 20, 31, 32]. This time limit was also provided by FLS curriculum.

Each of the recruited three volunteers performed three tasks. As a result, the mean time of the peg transfer task was 215 s with SD of 19 s and the surgical instruments were always in view during the task, as summarized in Table 2. According to Table 2, all volunteers succeeded to perform the modified peg transfer task within the 300 s, which is the time limit. To evaluate the effectiveness of the iNMI in more detail, a similar peg transfer task was performed by the three volunteers but they were not allowed to manipulate the iNMI and the MTM simultaneously—in order to mimic the swapping of control of the current surgical robot system. The results of the task are summarized in Table 2. As can be seen in Table 2, execution times of the tasks were evidently increased and one volunteer failed to finish the task within the time limit of 300 s. Furthermore, in this case, the surgical instruments were at times out of sight.

**Table 2:** Execution time of modified peg transfer task

Trial Number	Volunteer 1	Volunteer 2	Volunteer 3	Total mean
Allow simultaneous operation				
1	285	223	204	237
2	263	187	197	216
3	198	185	191	191
Mean	249	198	197	215
SD	37	17	5	19
Forbid simultaneous operation				
1	330	287	295	304
2	308	289	271	289
3	286	254	289	276
Mean	308	277	285	290
SD	18	16	10	11

*SD* standard deviation

## Discussion

To enable continuous surgical operation by enabling simultaneous manipulation of the 4-DOFs ECS and PSMs, two iNMIs were installed to two MTMs of the dVRK system.

To estimate the accuracy of the iNMIs, the accuracy test was designed and repeated 50 times where no error was detected. The results of the data transfer time and power consumption experiments indicate that the motions of the proposed ECS are in accordance to the surgeon’s intent in 5 ms via the iNMIs. This can be considered as real time [33], and the power capacity is sufficient for various kinds of surgeries. Moreover, since the power source of the iNMI can be simply replaced, the iNMI is still effective for those surgeries which exceed power volume of the iNMI. These experimental results indicate that the iNMI is able to precisely reflect the intent of surgeon and manipulate the 4-DOFs ECS without errors. The iNMI is currently under plan to be directly integrated with MTM of dVRK and communicate with the controller based on wired communication protocol. In this case, the effectiveness and utility of the iNMI is expected to be increased further as the problem of battery consumption and latency will be alleviated.

The developed simple 3D endoscope provides 3D vision by processing the acquired images using a stereo calibration and rectification algorithms. The volunteers recruited for the experiments used the stereo viewer, which displays two stereo images each on its left and right monitors, to obtain 3D vision during the task. All volunteers were able to determine distances and depths of objects on the provided view and execute the experiments.

As illustrated in Fig. 9, the workspace of the 4-DOFs ECS was calculated using its joint information. The shape of the estimated workspace, which is a cone-like shape, indicates the fulcrum point motion of the system. This was stably accomplished by its hardware structure rather than a software algorithm. Therefore, the proposed system provides a reliable and steady fulcrum point motion during robotic surgeries. The 4-DOFs ECS can be used for various kinds of surgeries which have smaller workspaces than that of the cholecystectomy since the system’s workspace is much larger than the cholecystectomy workspace. In addition, the calculated workspace can be further increased by adjusting the scale of the 4-DOFs ECS because it is manufactured with a relatively small size compared with the PSM. The resulting mean times of the modified peg transfer tasks were within the time limit of the FLS peg transfer task, even when the longer peg transfer board was used for the experiments in order to involve endoscope movements. This demonstrates that good performance is provided by the proposed surgical robot system using the iNMI, 4-DOFs ECS, and simple 3D endoscope. The gradually decreased execution time of each modified peg transfer task, as shown in Table 2, can be inferred as the volunteers were able to rapidly adapt to the system. In addition, the execution time of the peg transfer task and the number of surgical instruments obscured from view demonstrably increased when simultaneous operation of the MTM and the iNMIs was not possible. This indicates that the discontinuous surgical flow would indeed result in longer surgical operation time and collisions between instruments or injury to patients caused by the surgical instruments being obscured from view.

Using the proposed system, consisting of the iNMI based on HOTAS concept, simple 3D endoscope, 4-DOFs ECS, and dVRK system, surgeons will be able to simultaneously operate the PSM with the 4-DOFs ECS, and thereby ensure continuous surgical flow, which will result in safer robotic surgery environments and decreased operation time. The current 4-DOFs ECS can be only used as research purpose because of its relatively small size compared with actual endoscope system. However, the size of the system can be simply scaled-up to be used for real surgery. The sealing of 4-DOFs ECS is planned to be performed in the future for solving sterility issue [34].

## Conclusions

Robot-assisted laparoscopic surgery has high necessity since it provides various advantages compared with open surgery and conventional MIS. However, one problem with robotic surgery is the discontinuous surgical flow that occurs when control is being switched between the endoscope system and the PSM to change view and manipulate tools. This leads to problems such as possibility of injury to patients as a result of surgical instruments being out of sight, collision between surgical instruments, increased operation time, and enduring of unsatisfactory view to avoid control switching. To rectify these problems, this paper presented a wireless controller to simultaneously control the proposed endoscope system consists of a 4-DOFs ECS and a simple 3D endoscope. The 4-DOFs ECS uses four servo motors to generate pitching motion, yawing motion, rolling motion, and translational motion. The fulcrum point motion, which is necessary for the laparoscopic surgical robot system, is achieved by adopting a two-parallel link structure. The workspace of the 4-DOFs ECS is shown to have clinical applicability. The simple 3D endoscope, which has a diameter of 10 mm, was developed using two CMOS camera modules with six built-in LEDs. The two images acquired by the image sensors undergo stereo calibration and rectification to provide a clear stereo vision, and reconstructed stereo images are provided to the user via the stereo viewer of the dVRK system to enable 3D vision during surgery. The design of the proposed wireless master interface was based on the HOTAS concept, called iNMI, enables simultaneous manipulation of the 4-DOFs ECS and the operation robot arm. The results of power consumption tests, latency experiments, and accuracy tests demonstrate that the proposed iNMI is practicable and effective. Further, the results of a modified peg transfer task indicate that the proposed system is able to provide continuous surgical operation and therefore remove the issues affecting surgical robot systems. The size of the 4-DOFs ECS can be simply scaled-up for being used in clinics and the sealing issue will have to be resolved in order to overcome sterility issue.

## Abbreviations

MIS: minimally invasive surgery
DOFs: degrees of freedom
PSM: patient side manipulator
dVRK: da Vinci research kit
ECS: endoscope control system
3D: three-dimensional
iNMI: improved novel master interface
MTM: master tool manipulator
CMOS: complementary metal–oxide–semiconductor
HOTAS: hands-on-throttle-and-stick
LED: light-emitting diode
RC: resistor–capacitor
SD: standard deviation
FLS: fundamentals of laparoscopic surgery
